# In vivo porcine characterization of atrial lesion safety and efficacy utilizing a circular pulsed‐field ablation catheter including assessment of collateral damage to adjacent tissue in supratherapeutic ablation applications

**DOI:** 10.1111/jce.15522

**Published:** 2022-05-16

**Authors:** Jonathan C. Hsu, Douglas Gibson, Rajesh Banker, Shephal K. Doshi, Brett Gidney, Tara Gomez, Dror Berman, Keshava Datta, Assaf Govari, Andrea Natale

**Affiliations:** ^1^ Cardiac Electrophysiology Section, Division of Cardiology, Department of Medicine University of California, San Diego San Diego California USA; ^2^ Interventional Electrophysiology Scripps Clinic and Prebys Cardiovascular Institute La Jolla California USA; ^3^ Premier Cardiology Newport Beach California USA; ^4^ Pacific Heart Institute Santa Monica California USA; ^5^ Heart Rhythm Center Santa Barbara California USA; ^6^ Biosense Webster Inc. Irwindale California USA; ^7^ Texas Cardiac Arrhythmia Research Austin Texas USA; ^8^ MetroHealth Medical Center Case Western Reserve University School of Medicine Cleveland Ohio USA

**Keywords:** catheter ablation, irreversible electroporation, preclinical model, pulmonary vein isolation, pulsed‐field ablation

## Abstract

**Introduction:**

Pulsed‐field ablation (PFA), an ablative method that causes cell death by irreversible electroporation, has potential safety advantages over radiofrequency ablation and cryoablation. Pulmonary vein (PV) isolation was performed in a porcine model to characterize safety and performance of a novel, fully‐integrated biphasic PFA system comprising a multi‐channel generator, variable loop circular catheter, and integrated PFA mapping software module.

**Methods:**

Eight healthy porcine subjects were included. To evaluate safety, multiple ablations were performed, including sites not generally targeted for therapeutic ablation, such as the right inferior PV lumen, right superior PV ostium, and adjacent to the esophagus and phrenic nerve. To evaluate the efficacy, animals were recovered, followed for 30(±3) days, then re‐mapped. Gross pathological and histopathological examinations assessed procedural injuries, chronic thrombosis, tissue ablation, penetration depth, healing, and inflammatory response.

**Results:**

All eight animals survived follow‐up. PV narrowing was not observed acutely nor at follow‐up, even when ablation was performed deep to the PV ostium. No injury was seen grossly or histologically in adjacent structures. All PVs were durably isolated, confirmed by bidirectional block at re‐map procedure. Histological examination showed complete, transmural necrosis around the circumference of the ablated section of right PVs.

**Conclusion:**

This preclinical evaluation of a fully‐integrated PFA system demonstrated effective and durable ablation of cardiac tissue and PV isolation without collateral damage to adjacent structures, even when ablation was performed in more extreme settings than those used therapeutically. Histological staining confirmed complete transmural cell necrosis around the circumference of the PV ostium at 30 days.

## 1 INTRODUCTION

Catheter ablation for the treatment of atrial fibrillation is a well‐established procedure that is typically performed with radiofrequency (RF) or cryothermic energy sources, which destroy target tissue by heating or freezing, respectively.^1^ Despite continuing advances in ablation technologies for lesion efficacy, the use of these energy sources is still associated with rare but potentially serious complications resulting from injury to targeted and adjacent tissues, such as pulmonary vein (PV) stenosis, phrenic nerve palsy, and atrio‐esophageal fistula.^1^, ^2^ Various procedural techniques are currently employed with the aim of minimizing the risk of these complications,^1^, ^3^ but the challenge of balancing ablation effectiveness and safety remains.

Pulsed‐field ablation (PFA) is an ablative method that employs high voltage very‐short duration pulses that result in the destabilization of cellular membranes, via the formation of pores in the cytoplasmic membrane and death by a mechanism of irreversible electroporation.^4^, ^5^, ^6^, ^7^ In contrast to established thermal energy modalities, which ablate any tissue with which they are in contact, PFA has the potential to be more tissue‐specific owing to differences in threshold field strengths that induce cell death in selected tissues, with cardiomyocytes having one of the lowest threshold values of any tissue.^8^, ^9^, ^10^, ^11^

The higher selectivity of PFA tailored for cardiac ablation is expected to reduce the risk of inadvertent injury of adjacent anatomical structures and thus provide the much‐desired improvement in the safety of ablation. Initial preclinical studies have confirmed that PFA can produce transmural and durable cardiac lesions with minimal effect to the esophagus, phrenic nerve, or the coronary arteries.^12^, ^13^, ^14^, ^15^, ^16^, ^17^ These studies also demonstrated an improved risk‐benefit profile with PFA compared with RF ablation and cryoablation, providing further evidence that this technique could help to optimize outcomes in the clinical setting.^12^, ^17^ So far, the clinical use of PFA in small numbers of patients with AF has not shown any safety concerns.^18^, ^19^, ^20^

**Figure 1:**
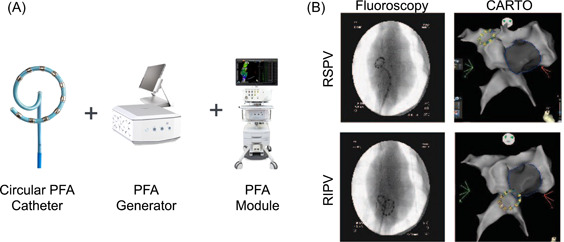
Biosense Webster PFA system. (A) Components of the Biosense Webster PFA system. (B) Circular PFA catheter deployed in the RSPV and RIPV visualized on fluoroscopy and CARTO images. PFA, pulsed field ablation; RIPV, right inferior pulmonary vein; RSPV, right superior pulmonary vein.

Amongst the studies published to date, most have used stand‐alone ablation catheters without mapping capabilities or integration with electroanatomical mapping systems. This study used a novel integrated PFA system including a PFA Generator, a PFA Circular Catheter, and a compatible mapping system (Figure 1). In a recent preclinical study, ablation with the PFA Circular Catheter in a porcine model was shown to provide durable right atrial ablation lines with a reduced risk of esophageal or phrenic nerve injury compared with RF ablation.^17^

The objective of this study was to characterize the performance of a novel PFA system, consisting of the PFA Generator, PFA Circular Catheter, and CARTO System (Biosense Webster), in providing pulmonary vein isolation (PVI) using an in vivo porcine beating heart model. To explore safety more fully with this system, we applied ablations directly over or in proximity to noncardiac tissues that can be vulnerable to collateral injury.

## 2 METHODS

### 2.1 Animals and protocols

The study included eight healthy porcine subjects studied under general anesthesia with isoflurane inhalation and mechanical ventilation. The study was performed at Global Medical Devices Laboratories, Inc (GMD Labs) (six animals) and Absorption Systems (two animals). Care and use of animals in this study were approved by the Institutional Animal Care and Use Committee (IACUC) at each of the study sites and conformed to the Position of the American Heart Association on Research Animal Use and the guiding principles of the Declaration of Helsinki.

### 2.2 Experimental design

The primary endpoints focused on safety assessments based on demonstration that ablations were created without collateral tissue injury and/or adverse thermal effects. This was determined via assessment of acute and 30 (±3)‐day follow‐up collateral tissue damage associated with ablation in both the right and left atria; assessment of electrical activity of cardiac tissue surrounding the ablation lesion; assessment of any thermal effects associated with ablation; and assessment of mechanical tissue damage associated with device use. The secondary endpoints focused on efficacy assessments based on demonstration that ablations created in clinically relevant anatomical regions were effective (i.e., permanent and durable) acutely and at chronic timepoints, via the assessment of lesion durability and characterization of lesion depth and width. Together, these endpoints were intended to characterize the safety profile of the PFA system when performing multiple overlapping ablations at a given location in the atria and when PFA applications were applied directly over or close to noncardiac tissue such as the PVs, phrenic nerve, and esophagus.

### 2.3 Ablation procedure

The ablation system studied included a PFA Circular Catheter and PFA Generator used with the CARTO Mapping system (Biosense Webster). The 7.5 Fr circular catheter includes 10 electrodes with individual irrigation pores (Figure 1). The circular catheter has an adjustable diameter between 25 mm and 35 mm which allows for the positioning of the catheter over a wide range of pulmonary vein ostia sizes. This ablation catheter was advanced to the targeted anatomic region using a steerable sheath (VIZIGO™, Biosense Webster). The catheter is bidirectional in handling with 180° deflection to one side and 90° to the other to facilitate catheter engagement to all PVs. PFA is applied in a bipolar configuration between skipped electrodes (i.e., electrode 1 to electrode 3) and between each of the adjacent electrodes between them (i.e., electrodes 1–2 and 2–3). Each PFA application includes trains of biphasic pulses between all three bipolar configurations for a total application duration of approximately 250 ms. Notably, there was no time delay between applications, with the aim of testing more extreme settings than those used therapeutically. A constant irrigation flow rate of 4 ml/min is maintained during the procedure. All procedures were performed under anticoagulation with heparin and activated clotting time range of 300–400 s. An ablation parameter setting of 1800 V on the PFA generator for 10 electrodes was used.

A total of 16 ablations were targeted for each of the targeted cardiac structures to evaluate the safety of delivery of supratherapeutic energy levels to these tissues (Table 1). Assessments were performed in both the left and right atrial chambers. One PV per animal was targeted for isolation by delivering ablation to the ostium, while a second PV was targeted for intensive narrowing stress (stenosis), by ablating directly deep inside of the vein. In the right atrium, the phrenic nerve was mapped before ablation with pacing from the mapping catheter to elicit diaphragmatic contraction from phrenic nerve capture, and ablations were delivered directly adjacent to this area near the phrenic nerve. After ablation, the phrenic nerve was paced for phrenic nerve capture. The lateral mitral valve was targeted from the left atrium; left ventricular ejection fraction was assessed postablation as an indicator of valve damage related to the procedure. In addition, the aorta and esophagus adjacent to the heart were each independently mapped, and ablations were delivered directly into the aorta wall due to the fact that it is closest anatomically to the esophagus. Following the ablation procedure, animals were recovered and followed for 30 (±3) days and then were mapped again before being euthanized for gross pathological and histopathological examination.

**Table 1:** Ablation parameters.

Target anatomic region		Number of active electrodes	Rationale for number of active electrodes	Target Number of applications
Left atrium	RSPV	6–10	Circumferential	16
	RIPV	6–10	Circumferential	16
	Roof	6–10	Segmental	16
	Appendage	6–10	Circumferential	16
	Lateral mitral valve	3–6	Segmental	16
Right atrium	SVC	All 10	Circumferential	16
	Posterior Wall	3–10	Linear/line	16
Aorta	Proximity to esophagus	6–10	Circumferential	16

Abbreviations: RIPV, right inferior pulmonary vein; RSPV, right superior pulmonary vein; SVC, superior vena cava.

### 2.4 Histopathological analysis

Triphenyl tetrazolium chloride was infused 15 min before euthanasia to facilitate the differentiation between metabolically active and inactive tissue; this redox indicator stains viable tissue red while the nonviable tissue remains white. Following euthanasia, the heart–lung–esophagus complex including the phrenic nerve were dissected and excised en bloc. After documentation of any abnormalities or injuries, tissues were preserved in 10% neutral buffered formalin for histological analysis. Following fixation, all affected regions of the right atrium, right atrial appendage, superior vena cava (SVC), right superior pulmonary vein (RSPV), right inferior pulmonary vein (RIPV), and the aorta were trimmed at 2‐mm intervals and embedded in paraffin for slide preparation. All tissues were stained with hematoxylin‐eosin (H&E), while the heart sections were additionally stained with Mason's Trichrome. The tissues were then assessed for procedural injury, chronic thrombosis, tissue ablation, depth of penetration and healing, and level of inflammatory response.

### 2.5 Statistical analysis

Study findings were analyzed and presented using descriptive statistics. Presented data for categorical variables included the number in each category; for continuous variables, the results included means and standard deviations and were compared using one‐way analysis of variance (ANOVA) and Tukey's pairwise test, as appropriate. Statistical analyses were performed with GraphPad Prism software version 9.0.0 (GraphPad Software).

## 3 RESULTS

### 3.1 Animals and procedural outcomes

Each of the 8 animals received PFA treatment. All eight animals survived for the 27–30–day follow‐up period. There was no occurrence of thrombus and/or charring on the catheter tip, pericardial effusion and/or cardiac tamponade, steam pop events, or mural thrombus (on intracardiac echocardiography [ICE] during the procedure nor during gross pathology), no incidence of clinically significant mechanical tissue injury was noted from gross pathology, and no incidence of clinically significant thrombo‐emboli was found in upstream and downstream organs nor within the heart.

All animals were in sinus rhythm, except for one, which was in sinus tachycardia. The sinus tachycardia coincided with low blood pressure during the remap procedure and general instability and, as such, was deemed by the physician as a remapping procedure related complication. No myoglobin was found in urine samples drawn at 30 days postablation, indicating the absence of skeletal muscle damage.

### 3.2 Assessment of pulmonary vein narrowing

**Figure 2:**
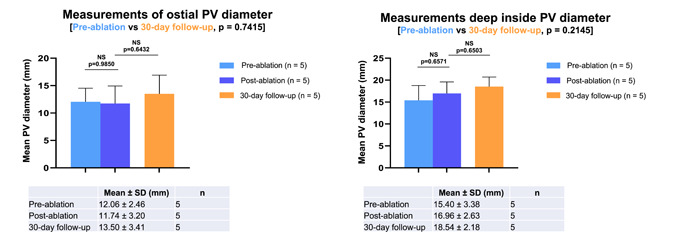
Mean RSPV and RIPV diameters^a^ after treatment with PFA. ^a^PV measurements were performed for all animals as per the protocol, but some values were missing from the analyses due to loss of ultrasound or fluoroscopy images at the laboratory; five animals had deep vein assessment. Statistical tests performed include one‐way ANOVA and Tukey's pairwise tests for comparisons between groups. ANOVA, analysis of variance; PFA, pulsed‐field ablation; PV, pulmonary vein; RIPV, right inferior pulmonary vein; RSPV, right superior pulmonary vein; SD, standard deviation

There were no incidents of PV narrowing on the same day (pre‐ vs postprocedure PV diameters) or 30 days post PFA application (pre‐ or postprocedure vs 30‐day follow‐up diameters) when the vein was targeted for isolation via ablation delivered to the ostium, nor when ablation was performed directly inside of the vein deep to the ostium, as demonstrated by X‐ray, ICE, and by flow velocity data (Figure 2).

### 3.3 Effect of myocardial PFA procedure on the various adjacent ablated tissue types

**Figure 3:**
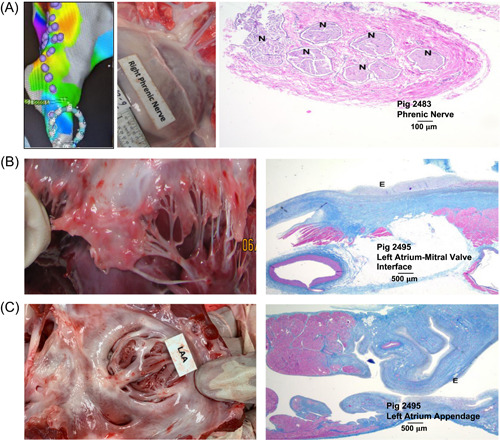
Effect of PFA procedure on adjacent cardiac tissues. (A) CARTO map showing position of phrenic nerve, gross pathology at 30 days postablation showing no tissue damage, and Masson's trichrome and H&E stain at 30 days postablation showing structural integrity of phrenic nerve; (B) Gross pathology and histology of mitral valve at 30 days postablation showing no tissue damage; (C) Gross pathology and histology of left atrial appendage at 30 days postablation showing no tissue damage. H&E, hematoxylin‐eosin; LAA, left atrial appendage; PFA, pulsed‐field ablation

The phrenic nerve was mapped before ablation, such that ablations could be delivered directly adjacent to the phrenic nerve on the endocardial aspect. No acute injury was seen, nor was any injury found during gross pathology (Figure 3A).

The mitral valve was explicitly targeted with at least four applications of energy in all treated animals. As a safety assessment, valvular function was evaluated via ICE and physician assessment before the procedure, directly after the procedure, and at 30 days. No valvular damage was noted (Figure 3B). Additionally, in gross pathology, the functional components of the valve were all intact. Left ventricular ejection fraction (LVEF) was assessed in 6 of the 8 animals pre‐ablation, directly after the procedure, and at 30 days postprocedure, and showed no deterioration of ventricular function over the follow‐up period; in the six animals assessed for ventricular function, LVEF ranged from 69%‒89% pre‐ablation, 69%‒86% immediately after ablation, and 59%‒86% at 30‐day follow‐up, as shown in Figure S1. No damage was observed in the left atrial appendage (Figure 3C).

**Figure 4:**
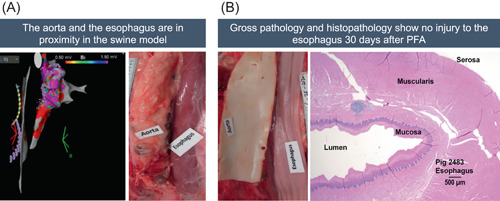
Effect of PFA procedure on adjacent esophagus. (A) CARTO map showing that the anatomic position of esophagus and aorta are in close proximity, also apparent during gross pathology; (B) Gross pathology at 30 days after ablation delivered to the aorta wall showing no damage to the adjacent esophagus, even when indentations on the aorta are present indicating some mechanical pressure applied during original energy applications, with Masson's trichrome and H&E stain of esophagus section 30 days postablation showing structural integrity. H&E, hematoxylin‐eosin; PFA, pulsed‐field ablation

In porcine subjects, the aorta and the esophagus are in close proximity to each other (Figure 4). The esophagus was targeted by delivering energy directly into the aorta wall. The esophagus was mapped, and between 11 and 21 applications of energy were delivered where the aorta was within <2 mm from the esophagus by anatomic adjacency. In three of the eight subjects, approximately 2 mm pits, which aligned with the placement and spacing of the catheter electrodes, were noted on the endothelium of the aorta during gross pathology, but no damage was seen in the directly adjacent esophagus. In all animals, no injury was seen grossly or histologically in the adjacent esophagus (Figure 4).

### 3.4 Circumferential pulmonary vein lesions

**Figure 5:**
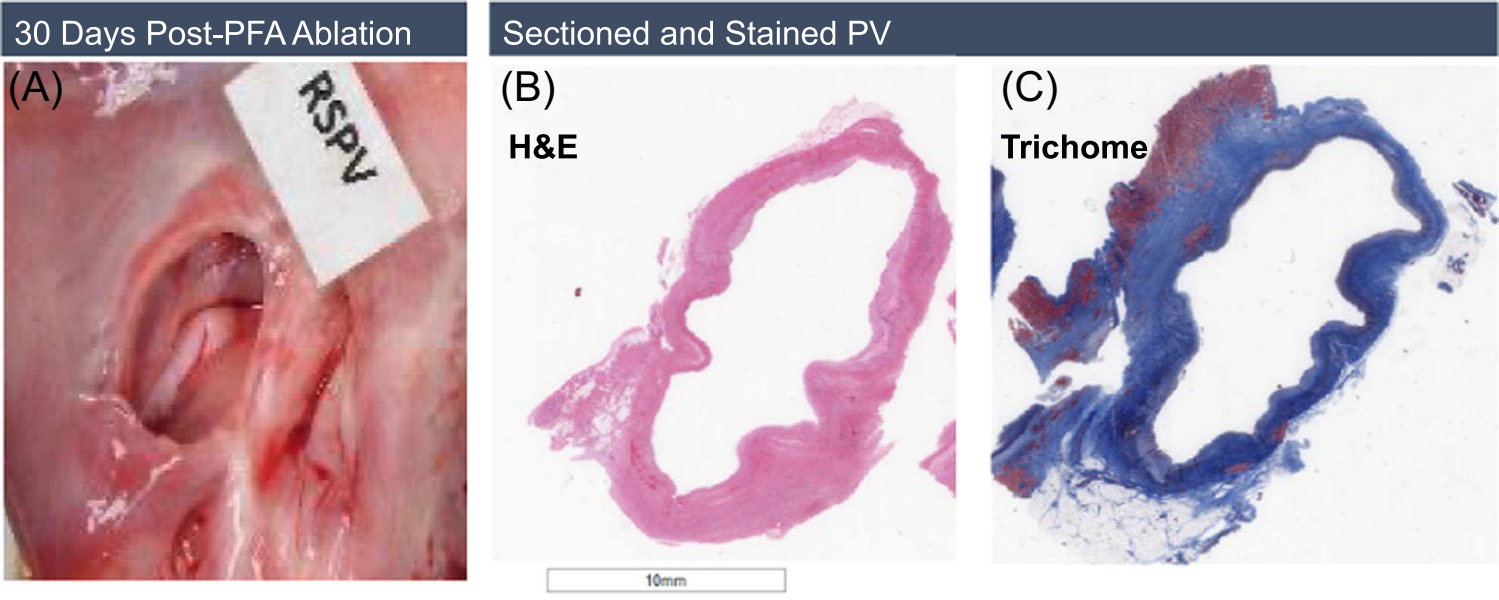
Contiguous circumferential lesions made at the RSPV. From left to right: RSPV orifice is covered with yellowish‐white scar tissue; H&E and Masson's trichrome stained section of the RSPV, with blue color indicating fully circumferential ablation and fibrosis/fibroplasia. H&E, hematoxylin‐eosin; PFA, pulsed‐field ablation; PV, pulmonary vein; RSPV, right superior pulmonary vein

Each heart was dissected longitudinally along the long axis of the ventricular and atrial free walls and opened to view the pulmonary veins, from which 3–5 sections were taken. On gross examination, it was noted in many animals that the PVs had a prominent confluent lesion encircling the entire orifice of the vein (Figure 5A). In particular, the RSPV sustained contiguous and transmural treatment‐related necrosis and repair. The endocardial borders of the veins were fully remodeled circumferentially and showed replacement of the myocardial tissue with fibrous connective tissue. The medial layer underneath had a similar chronic effect exemplified by the proliferation of fibro‐elastic elements mixed with variable collagen matrix. Histological examination showed complete, transmural necrosis around the entire circumference of the ablated section of the RSPV (Figures 5B,C).

### 3.5 Durability of ablation

**Figure 6:**
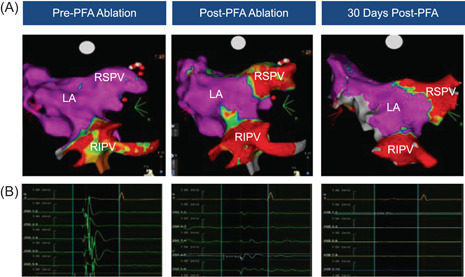
Durability of electrical isolation with the PFA procedure. (A) Pre‐PFA voltage map and post‐PFA voltage maps acquired directly after the first procedure and after 30 days of follow up for re‐mapping; (B) EGMs taken before PFA, directly after PFA, and 30 days after the procedure during a re‐mapping procedure, showing acute electrical isolation with persistence of isolation in follow‐up. EGM, electrogram; PFA, pulsed‐field ablation.

Eliminations of PV potentials were confirmed in all animals using the Pentaray diagnostic mapping catheter both directly after the initial ablation procedure and at the 27–30–day follow up before euthanasia (Figure 6). Similarly, for ablation procedures in the right atrium, electrogram voltage reduction was confirmed following the initial ablation procedure in all animals. After 30 days, chronic scarring was still visible along the posterior wall consistent with the original sites of ablation. Confirmation of elimination of PV potentials in the targeted PV at 30‐day follow up before euthanasia was confirmed in all animals.

## 4 DISCUSSION

This preclinical evaluation of a PFA system demonstrated effective and durable ablation of cardiac tissue with no collateral damage to adjacent structures, such as the phrenic nerve, esophagus, or mitral valve, even when these structures were deliberately targeted by more extreme ablation applications than would be used therapeutically.

These results are consistent with and expand on findings from previous preclinical studies of PFA. A recent study using the PFA Circular Catheter and multichannel generator to deliver right atrial ablation line of block in 12 porcine subjects demonstrated durable atrial lesions, with the line remaining blocked after 28 days of survival in 11 animals and transmural fibrosis demonstrated on pathological examination.^17^ In six animals who had PFA delivered from the RIPV close to the esophagus, there was no evidence of injury to the esophageal adventitia or mucosa on postmortem inspection; in contrast RF ablation in three other animals resulted in acute esophageal injury. Similarly, PFA delivered over the course of the phrenic nerve in 10 porcine subjects was associated with no clinical signs of functional nerve injury, while RF ablation in two animals was associated with acute phrenic nerve paralysis lasting for more than 30 min after ablation.^17^ The results of our current study demonstrated the durability of PVI lesions in the porcine left atrium made with PFA over the 30‐day follow‐up, as demonstrated by voltage mapping. The durability of ablation lesions seen in the current study with histopathologic analysis, along with previous preclinical studies in this field, support the irreversibility of cardiac tissue death with the PFA procedure.

The current study demonstrated complete circumferential ablation of the PVs by gross and histological examination. Histological staining was particularly striking in showing the full extent of cell necrosis around the circumference of the PV at 30 days after ablation. It has been suggested that circumferential lesions with a higher degree of necrosis compared with edema postablation are associated with a lower risk of AF recurrence and that ablation strategies that provide this higher degree of necrosis may minimize the reversibility of myocardial injury and thus arrhythmia recurrence.^21^

Regarding safety of PFA, our investigation built on the findings of the previous preclinical model by employing ablation under more “extreme” conditions, such as ablation inside the PV lumen and directly to the mitral valve, as well as multiple applications at each site. The current study showed no evidence of PV stenosis after PFA either at the proximal ostium or deep inside of the PV. Stenosis of the PVs is a recognized possible complication of RF ablation, with risk factors including delivery of energy inside the veins and use of excessive power during RF application.^22^ Periprocedural real‐time imaging, such as ICE, can be used to guide ostial isolation and power titration. Our findings are consistent with previous preclinical models of PFA inside the PV,^16^, ^23^ thereby demonstrating the large safety margin provided by PFA when ablating close to and even within the PVs; thus, it may simplify PVI procedures. In one of those preclinical studies, a canine model, the effects of PFA using a 9‐electrode circular array catheter or irrigated radiofrequency ablation (IRF) were evaluated in eight animals, with animals randomized to receive one of the ablation technologies applied to both superior PVs and the other to both inferior PVs. With monitoring via computed tomography (CT) angiography‐based three‐dimensional modeling, PFA was associated with significant reductions in the risk of PV stenosis compared with IRF. Furthermore, IRF was associated with damage to the vagus nerve and lung parenchyma, and esophageal dilation, while PFA did not cause injury at any of these sites.^23^

No damage to the phrenic nerve or esophagus was detected in the animals treated in this study. Damage to these closely adjacent structures is recognized as a potential complication of RF ablation; while rare, development of atrio‐esophageal fistula is a life‐threatening event.^1^, ^2^ In porcine subjects, the RIPV and aorta are adjacent to the esophagus. The previous study reported by Yavin et al. applied PFA at the RIPV,^17^ while our study ablated the aorta to achieve proximity to the directly adjacent esophagus. The ablations noted in the aorta indicated that close proximity to the esophagus had been achieved. Also, the ablations themselves appeared as imprints on the inner lumen of the aorta. Importantly, both models confirmed the safety of PFA in avoiding esophageal damage, regardless of the route taken to try to inflict such collateral damage, and further illustrate the cardiac selectivity of PFA.

Additionally, early investigations of PFA raised concerns around voltage‐induced muscle contractions,^7^ but our study showed no evidence of rhabdomyolysis due to muscle contraction and no signs of procedure‐related arrythmias.

The limitation of this investigation is inherent to a preclinical animal model with unknown correlations to humans and, specifically in the context of PV ablation, some differences and variability in PV structures and branching. These differences were accounted for in the present study by opting to target the superior (RSPV) and inferior (RIPV) branches that connect to the left atrium via the right ostium in the porcine heart. Further, there was no control group (eg, using radiofrequency ablation) included in the current study. In addition, no direct visualization of the brain of porcine subjects was performed to assess for silent cerebral ischemia. Regarding the applicability of PFA utilizing this system in humans, the inspIRE clinical study (NCT04524364) is currently investigating the safety and effectiveness of PVI using the Circular PFA Catheter and PFA Generator in patients with paroxysmal atrial fibrillation.

## 5 CONCLUSION

In conclusion, an integrated PFA system encompassing a generator, catheter, and mapping module produced durable ablation lesions and circumferential ablation of the PVs with no esophageal, phrenic nerve, or mitral valve damage, even when ablation was performed in more extreme settings than those used therapeutically.

## Supporting information

Supplemental Figure 1. Left ventricular ejection fraction.

## Data Availability

Data supporting the findings of this study are available from the corresponding author on reasonable request.
