# Supplementary figures and images for: In vivo porcine characterization of atrial lesion safety and efficacy utilizing a circular pulsed‐field ablation catheter including assessment of collateral damage to adjacent tissue in supratherapeutic ablation applications
Source: J Cardiovasc Electrophysiol. 2022 May 16;33(7):1480–8. doi: 10.1111/jce.15522 (PMC9545022; doi:10.1111/jce.15522)

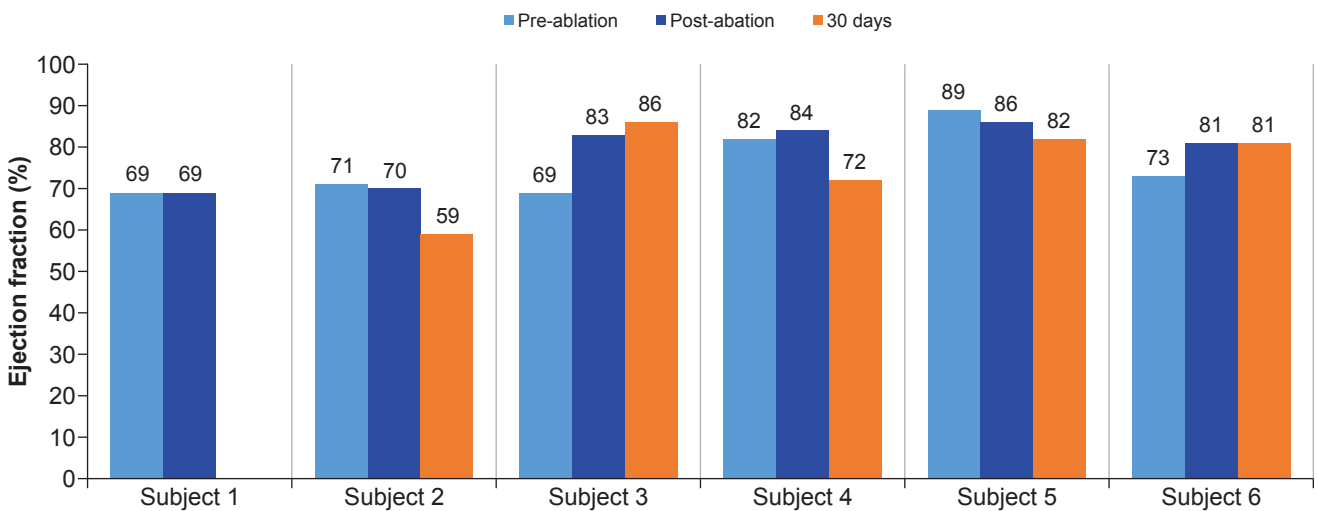

Supplement: Supplementary file 1 — Supplemental Figure 1. Left ventricular ejection fraction. [file JCE-33-1480-s001.pdf]
